# Structural Basis for MCAM Mediated Inhibition of Laminin Dependent Cell Migration

**DOI:** 10.64898/2026.09.11.751044

**Published:** 2026-09-18

**Authors:** Tongqing Li, Sofia Alarcon-Frias, Hengyi Li, Claudio R. Alarcón, Daryl E. Klein

**Affiliations:** a Department of Pharmacology, Yale School of Medicine, New Haven, CT 06510, USA; b Yale Cancer Biology Institute, Yale University, West Haven, CT 06516, USA

## Abstract

MCAM/CD146 is an immunoglobulin-superfamily receptor expressed on endothelial cells, immune cells and numerous carcinomas. Although MCAM has long been associated with metastatic progression, recent work has revealed context-dependent inhibitory effects on transendothelial migration, leaving unresolved how MCAM engagement of basement membrane laminins alters cell migration. Here, we report multiple cryo-electron microscopy structures of human MCAM bound to laminin a4. We show MCAM engages laminin on a surface centered on the first two laminin globular domains (LG1–2) and that this epitope directly overlaps with the interface used by α6β1 integrin. Consistent with structural competition, MCAM occupation of laminin α4 abolishes integrin-dependent cell migration. Structure-guided mutation of the MCAM-laminin interface disrupts laminin binding and eliminates MCAM-mediated inhibition. These findings establish that MCAM and integrin compete for a shared interface on laminin α4 and define a structural mechanism by which MCAM occupancy can suppress laminin-dependent migration. The structure resolves a central ambiguity in MCAM biology and provides a framework for developing biologics that selectively modulate pathological cell migration.

## Introduction

Metastasis remains the principal cause of cancer-related mortality and requires tumor cells to traverse vascular and stromal barriers during dissemination[1, 2]. A central step in this process is transendothelial migration, during which tumor cells cross the endothelial monolayer and penetrate the underlying basement membrane[3]. Basement membranes regulate adhesion, traction, survival and migration, thereby influencing whether cells can efficiently breach tissue boundaries[4, 5].

Laminins are heterotrimeric glycoproteins that form a major architectural and signaling scaffold of basement membranes[4, 5]. Laminins are composed of three chains (α, β, γ) and named accordingly (i.e. Laminin-αβγ). Distinct laminin isoforms confer different adhesive and migratory properties. Laminin-411 and laminin-511 are major components of endothelial basement membranes, with laminin α4-containing matrices generally associated with more permissive immune-cell and tumor-cell migration, whereas laminin α5-containing matrices can impose more restrictive barrier properties[6, 7]. These functional differences are particularly relevant at post-capillary venules, which are major sites of leukocyte extravasation and tumor-cell vascular transit[6–8].

Integrins are the principal force-generating receptors that enable cells to adhere to, remodel and migrate across lamininrich matrices[8, 9]. Laminin-binding integrins, including α6β1, engage laminin α globular domains and mechanically couple extracellular matrix engagement to the actin cytoskeleton[9, 10]. Recent structural work on α6β1 bound to laminin-511 established how the integrin headpiece recognizes a composite laminin surface involving the α-chain LG region and the γ-chain tail[10]. However, how other receptors engage α4-containing laminins, and whether those interactions are compatible with integrin binding, has remained unresolved.

MCAM, also known as CD146 or MUC18, is a type I trans-membrane immunoglobulin-superfamily receptor expressed by endothelial cells, pericytes, activated lymphocytes and multiple tumor types[11–14]. It was originally identified as a melanoma cell adhesion molecule (MCAM) and has been widely linked to melanoma progression, vascular interaction and metastatic potential[11–14]. Consistent with this view, MCAM has been described as a receptor that promotes tumor-cell adhesion, migration and invasion in several settings[12–14]. In immune biology, MCAM marks inflammatory T-cell subsets and binds laminin-411, facilitating entry of TH17 cells into the central nervous system[15]. MCAM and soluble MCAM have also been implicated in monocyte transendothelial migration and angiogenic signaling[16, 17].

Yet MCAM biology has remained controversial. In breast cancer, MCAM has been reported to suppress tumor-cell adhesion to endothelial cells and inhibit transendothelial migration[18]. Mannion and colleagues further emphasized that MCAM expression in tumors is heterogeneous and may reflect malignant cells, vasculature, normal epithelium or EMT-associated cell states, complicating interpretation of bulk expression data[18]. Thus, the field has lacked a molecular framework to explain how MCAM can be associated with pro-migratory phenotypes in some contexts while inhibiting migration in others. A critical unresolved question is whether MCAM and integrins cooperate on laminin substrates or instead compete for the same laminin surface. Previous biochemical studies showed that α6β1 integrin and MCAM bind α4-containing laminins and suggested that their binding sites lie near one another on the laminin α4 globular domain[19, 20]. However, no high-resolution structure has visualized MCAM bound to laminin α4, and no structural mechanism has explained whether MCAM binding is compatible with integrin engagement.

Here we define the MCAM-laminin α4 interaction by cryoEM and show that MCAM directly occludes the α6β1 integrin-binding surface. Functional and mutational analyses demonstrate that this structural interface is required for MCAM-mediated inhibition of laminin-dependent migration. These findings establish a direct competitive mechanism for MCAM and provide a unifying framework for interpreting context-dependent MCAM function in migration and metastasis.

## Results

### Cryo-EM structure of the MCAM-Laminin α4 complex

**Figure 1.**
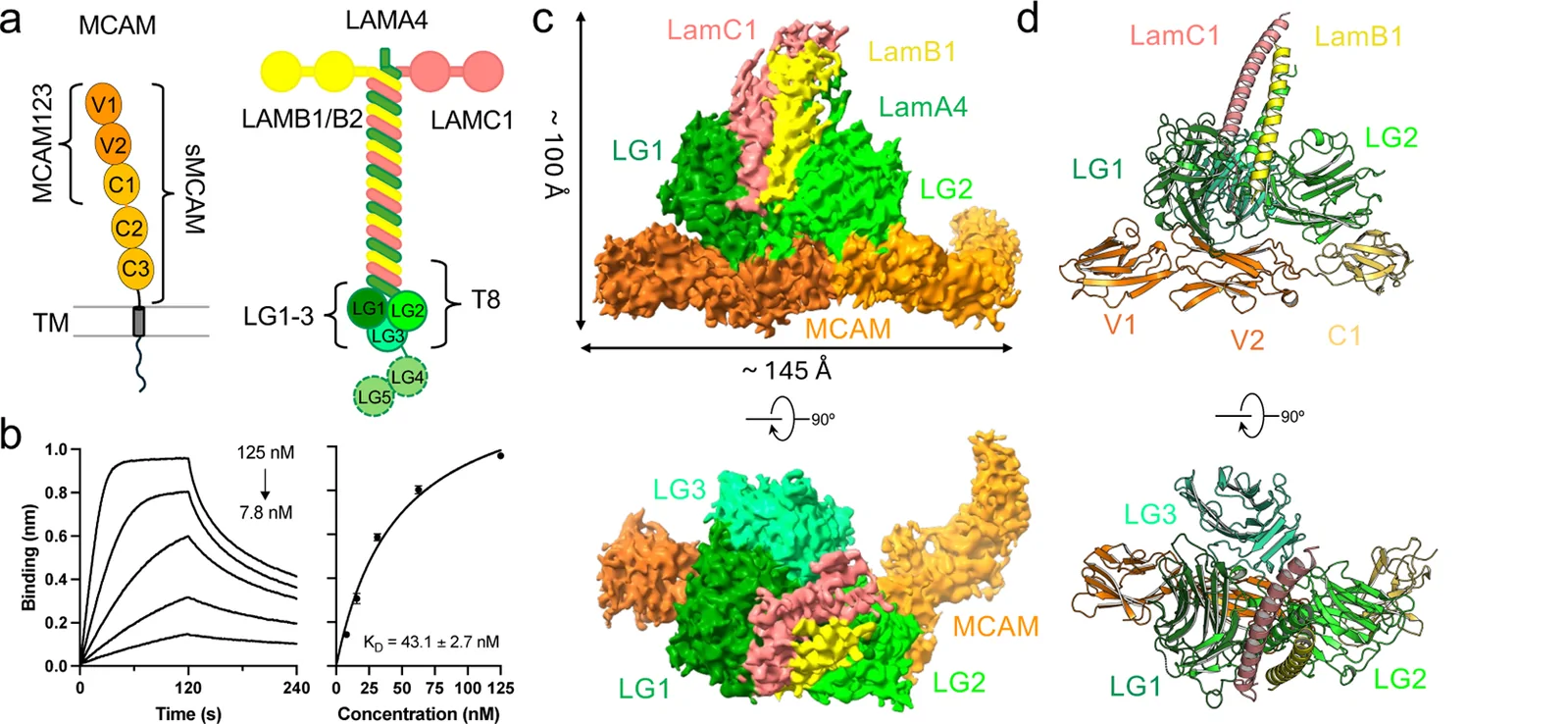
MCAM interacts with T8-LM411. **a**, Architecture scheme of MCAM and LamA4. The expression constructs used in this study are labeled by the side of curly brackets. **b**, Direct binding of T8-LM411to sMCAM detected by biolayer interferometry (BLI). The sensors were loaded with sMCAM. The binding affinity represents mean ± SEM from three independent experiment. **c**, Surface view of the Cryo-EM reconstruction of sMCAM-T8-LM411 complex (4.13 Å). **d**, Ribbon diagram of an atomic model of sMCAM-T8-LM411 complex. MCAM C2-C3 domains are not modeled as the resolution is over 4.4 Å (Supplementary Fig. 2).

To study the interaction between MCAM and LamA4, we purified multiple recombinant constructs of both proteins (Supplementary Fig. 1a). For LamA4 globular domain expression (LG1–3) we used either a construct that maintained a minimal heterotrimer coiled-coil designated T8[21], or the alpha chain’s globular domains in isolation without the beta or gamma chains (Fig. 1a). For MCAM expression we used a soluble construct (sMCAM, residues G26–G559) that comprises all five extracellular immunoglobulin (Ig)-like domains: two N-terminal V-type domains (V1, V2) followed by three C2-type domains (C1, C2 and C3) (Fig. 1a).

Biolayer interferometry (BLI) revealed that sMCAM binds T8-LM411 (LamA4: N757–E1405; laminin β1: D1714–L1786; laminin γ1: D1528–P1609) with an equilibrium dissociation constant (K_D_) of 43 nM (Fig. 1b), representing an approximately 40-fold stronger affinity compared to its interaction with LamA4 LG1–3 (residues Q833–E1405) (Supplementary Fig. 1b). This is consistent with prior observations that the laminin β and γ chains promote a more compact assembly by stabilizing the packing of LG domains[22], which may enhance MCAM binding affinity in solution.

**Figure 2.**
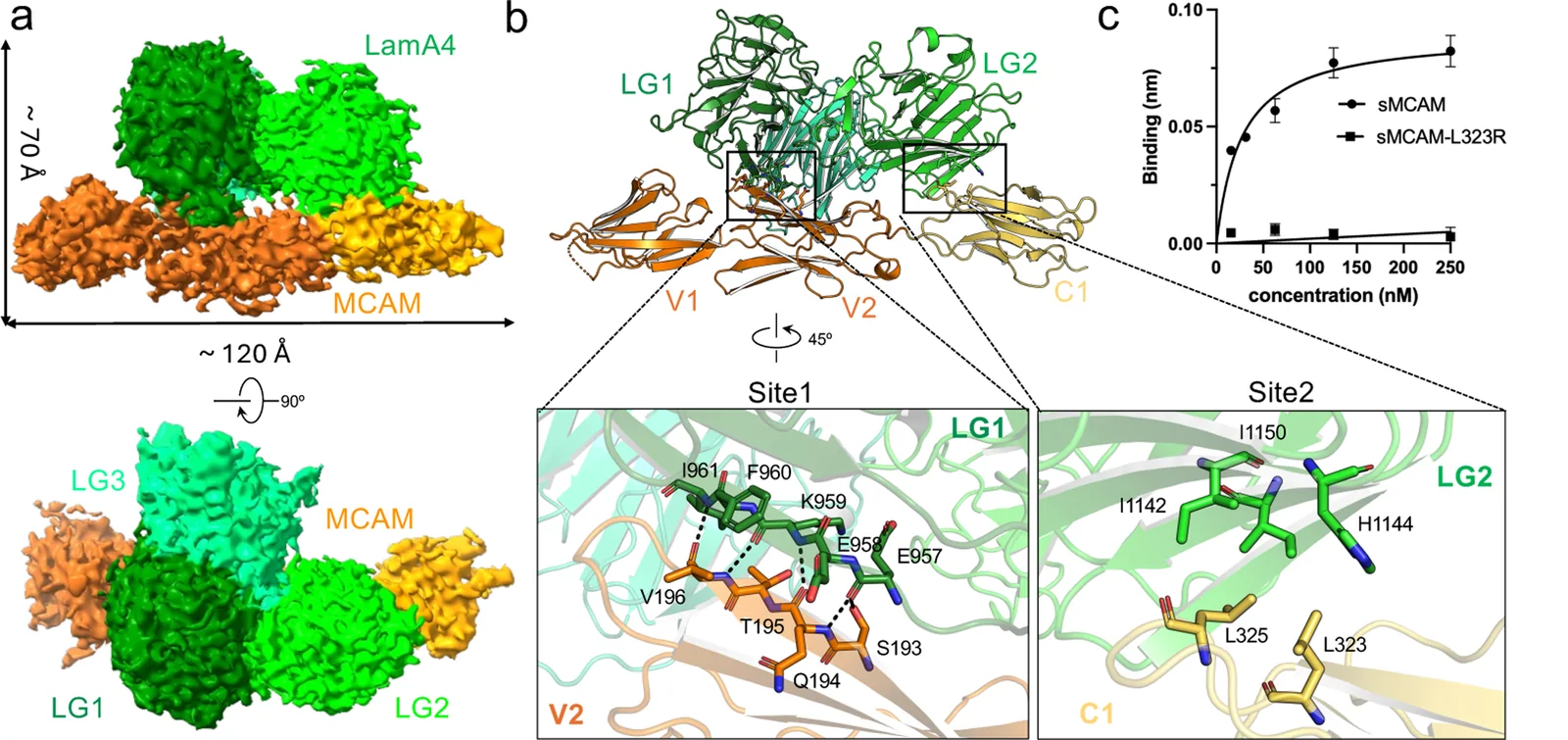
MCAM engages with LamA4 LG1–3 at two distinct sites. **a**, Surface view of the Cryo-EM reconstruction of the complex of sMCAM with isolated LamA4 LG1–3 domains (3.22 Å). **b**, Enlarged detailed view of site 1 interactions between MCAM V2 domain and LamA4 LG1 domain, and site 2 interactions between MCAM C1 domain and LamA4 LG2 domain. **c**, Binding of MCAM L323R mutant to T8-LM411 measured by BLI. The sensors were loaded with T8-LM411. The binding affinity represents mean ± SEM from three independent experiments.

For structural characterization, either T8-LM411 or LamA4 LG1–3 was mixed with sMCAM at a 1:1 molar ratio and subjected to cryo-EM (Supplementary Fig. 2 and 3). Image processing using cryoSPARC[23, 24] yielded a reconstruction at 4.13 Å resolution for sMCAM-T8-LM411 complex (Fig. 1c and d, Supplementary Fig. 2, Supplementary Table 1). Although the resolution is reduced due to flexibility in the laminin coiled-coil and distal sMCAM domains, the core involving MCAM and LamA4 interactions reaches a resolution better than 3.6 Å (Supplementary Fig. 2f). We also determined the cryo-EM structure of sMCAM in complex with LamA4 LG1–3 at 3.22 Å resolution (Fig. 2a and b, Supplementary Fig. 3, Supplementary Table 1). Both structures show the same over-all interaction. The complex adopts a compact architecture in which LamA4 LG1–3 forms a cloverleaf-like structure reminiscent of integrin-binding laminin fragments[22], while MCAM wraps along its surface. Two distinct interaction sites are observed. Site 1 is formed between the first globular domain of LamA4 (LG1) and the second Ig domain of MCAM (V2). Here, a series of main-chain interactions is made between MCAM (residues Q191–S198) and the V2 domain and LamA4 (residues T955–I961). Site 2 is formed between the second globular domain of LamA4 (LG2) and the third Ig domain of MCAM (C1). This interaction is dominated by hydrophobic contacts between MCAM’s L323 and LamA4 residues I1142 and I1150 (Fig. 2b, Supplementary Fig. 4a and b).

Mutational analysis supports the structural observations. Substitution of MCAM L323 with arginine abolishes binding to LamA4, while analogous mutations in LamA4 (I1142R or I1150R) weaken the binding affinities by about 5.9 fold for the I1142R mutant, and 2.7 fold for the I1150R mutant (Fig. 2b, Supplementary Fig. 4c). These results establish a bipartite binding mode in which hydrophobic interactions at site 2 are critical for complex stability.

**Figure 3.**
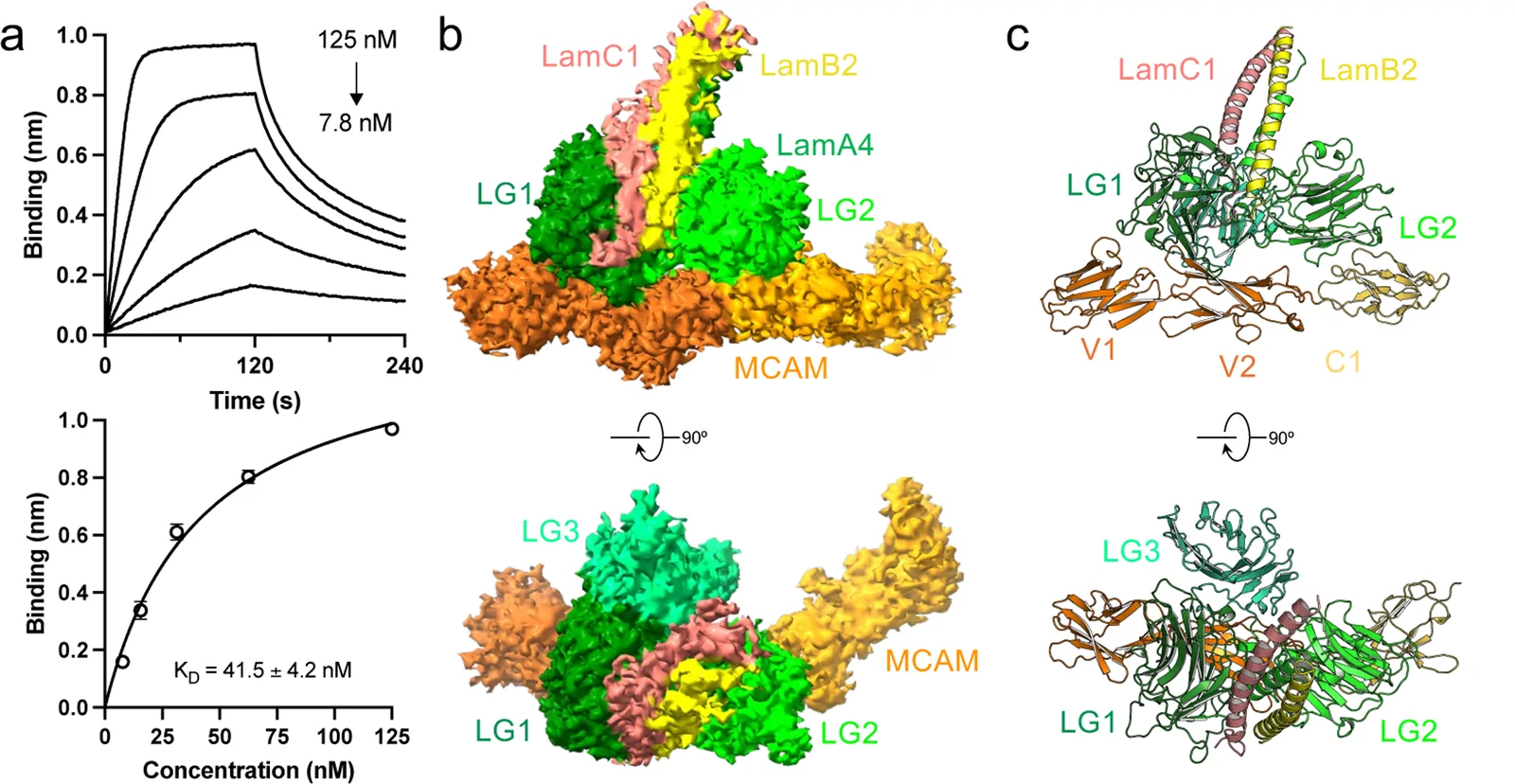
MCAM interacts with T8-LM421. **a**, Direct binding of T8-LM421 to sMCAM detected by BLI. The sensors were loaded with sMCAM. The binding affinity represents mean ± SEM from three independent experiments. **b**, Surface view of the Cryo-EM reconstruction of sMCAM-T8-LM421 complex (3.76 Å). **c**, Ribbon diagram of an atomic model of sMCAM-T8-LM421 complex.

Our structure indicates that MCAM engages exclusively with the laminin α4 chain. To investigate whether MCAM bindsLM411 and LM421 differently, we measured the binding of T8-LM421 (laminin β2: A1727–Q1798) to MCAM on BLI. The binding curves show a similar affinity (K_D_ = 41.5 nM) to that observed for T8-LM411 (Fig. 3a). We further determined a cryo-EM structure of MCAM in complex with T8-LM421 at 3.76 Å resolution (Fig. 3b and c, Supplementary Fig. 5). Similar to MCAM-T8-LM411, the laminin coiled-coil and distal MCAM domains of MCAM-T8-LM421 are flexible, but the core involving MCAM V2-C1 and LamA4 LG1–2 interactions is stable, with a resolution better than 3.3 Å (Supplementary Fig. 5e). Comparison of MCAM-LM411 and MCAM-LM421 complexes shows nearly identical binding interfaces, demonstrating that MCAM specifically recognizes the LG1 and LG2 domains of LamA4 and does not discriminate between the β1 and β2-containing isoforms LM411 and LM421.

### MCAM and Integrin α6β1 share overlapping binding sites on Laminin α4

**Figure 4.**
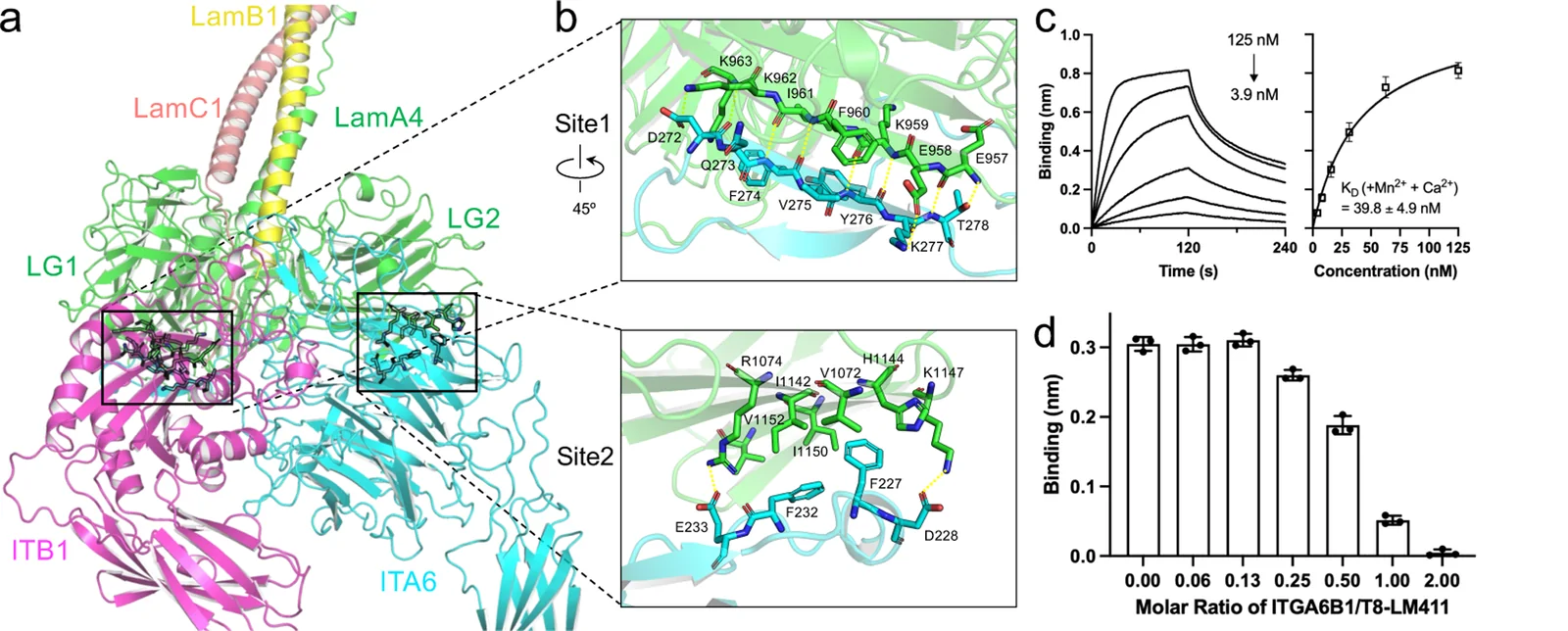
MCAM and Integrin α6β1 share overlapping binding sites on Laminin α4. **a**, AlphaFold 3 predicted complex model of T8-LM411 and integrin α6β1. The pLDDT and ipTM of the prediction is shown in Supplementary Fig. 5. **b**, Enlarged detailed view of site 1 interactions between integrin α6 and LamA4 LG1 domain, and site 2 interactions between integrin α6 and LamA4 LG2 domain. **c**, Binding of T8-LM411 to sMCAM in the buffer containing divalent metal cations detected by BLI. The sensors were loaded with sMCAM. The binding affinity represents mean ± SEM from three independent experiments. **d**, Competition between integrin α6β1 and sMCAM for LM411 binding measured by BLI. The sensors were loaded with sMCAM. 20 nM of T8-LM411 was mixed with increased concentrations of integrin α6β1. The responses at the plateau were plotted against the molar ratio of integrin α6β1 over T8-LM411. Bars represent mean ± SD from three independent experiments.

A prior structure of Integrin α6β1 bound to Laminin α5 (LamA5) revealed how integrins engage the laminin α-chain globular domains. However, there remains no experimental structure showing how Integrin α6β1 binds specifically to LamA4. Therefore, to make a direct comparison to our structure, we used structural modeling with AlphaFold 3[25] to predict the integrin α6β1 – T8-LM411 complex. The predicted complex is of high confidence (Supplementary Fig. 6a, ipTM = 0.79), and uses the same interface as the expericritical for complex stability. mental and highly similar LamA5 complex[10]. The α6β1-LamA4 interaction is mediated by residues that coincide with the MCAM-binding interface. Specifically, the LG1 β-sheet region (E957–K963; site 1) and hydrophobic residues in LG2 (including I1142 and I1150; site 2) are implicated in both MCAM and integrin binding (Fig. 4a and b, Supplementary Fig. 6a). Isoform-specific differences further support this model. Integrin α6X1β1 and α7X1β1 lack key elements required for site 1 interaction, suggesting partial overlap, whereas integrin α7X2β1 shows limited compatibility with the LamA4 interface, consistent with prior reports of weak or absent binding[9, 26, 27] (Supplementary Fig. 6b-d).

We experimentally tested this competition using BLI. Since integrin-laminin interactions are divalent metal cation-dependent[9, 10], we first confirmed that the presence of Mn^2+^ and Ca^2+^ does not affect MCAM–LamA4 binding (Fig. 4c). We then assessed whether integrin α6β1 could compete with MCAM for LM411 binding. Increasing concentrations of integrin α6β1 (ITGA6B1) progressively reduced T8-LM411’s ability to associate with sensor bound MCAM, demonstrating direct competition for overlapping binding sites (Fig. 4d). At a 2:1 ratio of ITGA6B1 to T8-LM411, no MCAM binding was detected, indicating that all MCAM binding sites were prebound with integrin.

### MCAM inhibits Laminin α4-mediated melanoma cell migration

**Figure 5.**
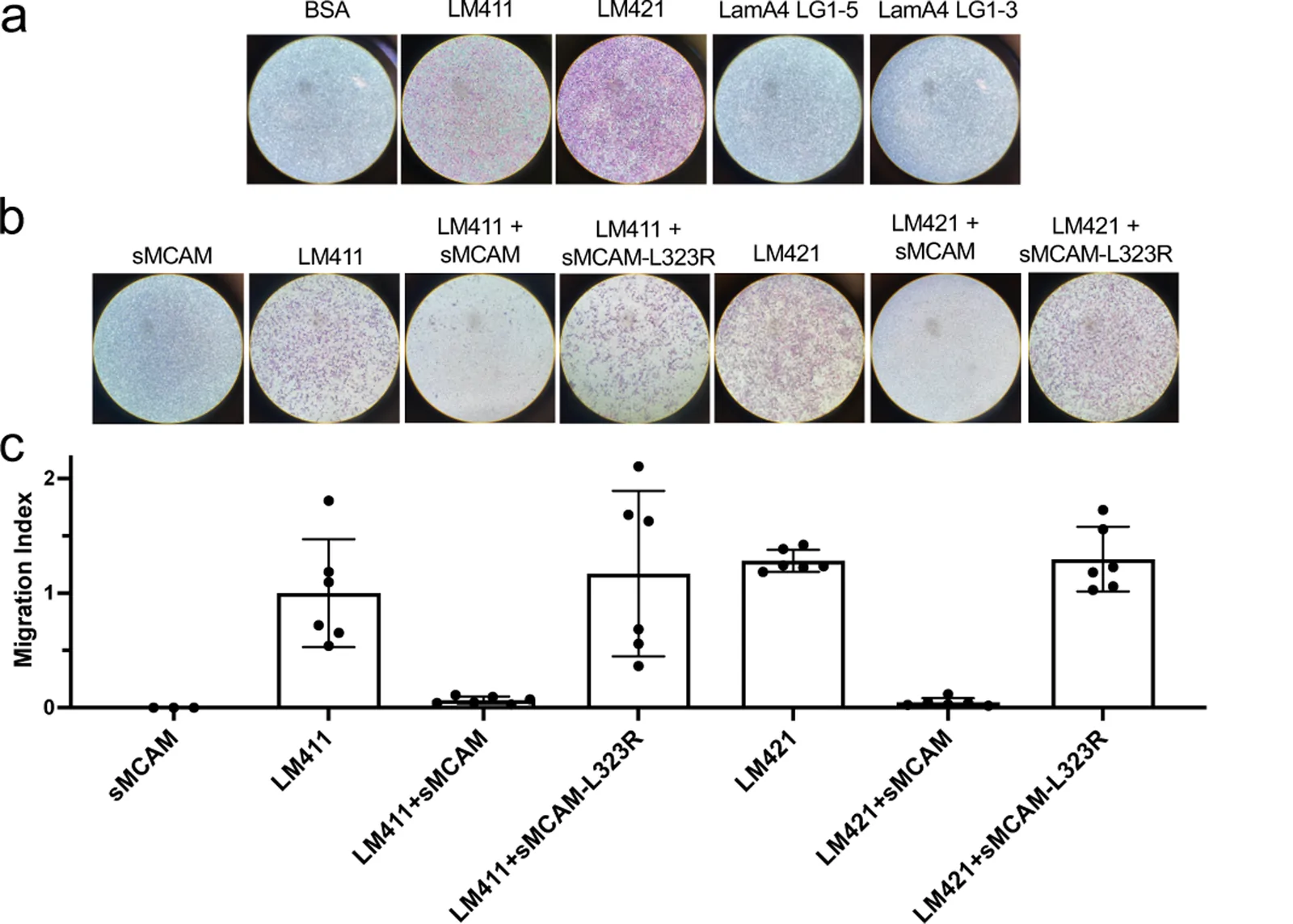
MCAM inhibits Laminin α4-mediated melanoma cell migration. **a**, LM411 and LM421 drive MeWo cell migration but not LamA4 LG domains. The concentration of the laminin proteins is 250 nM. **b**, sMCAM inhibits MeWo cell migration towards LM411 and LM421. The binding-deficient sMCAM-L323R mutant failed to inhibit the migration. The concentrations of LM411 and LM421 are 100 nM and 25 nM, respectively. The concentration of sMCAM and sMCAM-L323R is 1 μM. **c**, Quantification of the migration assays shown in **b**. Two transwell inserts were analyzed per condition, and each experiment was repeated three times for statistical analysis.

To evaluate the functional consequences of the MCAM-LamA4 interaction, we performed migration assays using MeWo melanoma cells. These cells exhibit robust migration towards both LM411 and LM421, with LM421 showing greater potency at comparable or lower concentrations (Fig. 5a, b), consistent with previous reports[19]. In contrast, isolated LamA4 LG domains failed to induce migration, indicating that intact laminin heterotrimers are required for this process (Fig. 5a). Given that MeWo cells express low levels of MCAM (Supplementary Fig. 7a), the observed migration is likely mediated by integrins rather than by MCAM. To exclude a major contribution from MCAM expressed on the cell surface, we performed the same assay using MCF7 breast cancer cells, which express low levels of both ITGA6 and ITGB1 compared to MeWo cells (https://www.proteinatlas.org/). MCF7 cells show little to no migration toward LM411 or LM421. Ectopic expression of MCAM in MCF7 cells does not enhance migration (Supplementary Fig. 7a and b), further demonstrating that laminin-dependent migration is driven by integrins and not MCAM.

Based on the shared binding interface, we hypothesized that MCAM could competitively inhibit integrin-dependent migration. Indeed, addition of sMCAM with LM411 or LM421 effectively blocked MeWo migration toward LM411 and LM421 (Fig. 5b and c). The inhibitory effect was recapitulated by the V1-C1 fragment of MCAM (MCAM123), indicating that the N-terminal domains are sufficient for competition (Supplementary Fig. 7d and e). In contrast, an in-house-generated Fab, which recognizes the LamA4 LG2 domain at an epitope distinct from the MCAM/integrin binding site (Supplementary Fig. 7c and 8), did not block MeWo migration (Supplementary Fig. 7d and e). Moreover, the binding-deficient MCAM L323R mutant failed to inhibit migration, confirming that blockade depends on direct interaction with LamA4 (Fig. 5b and c). These results demonstrate that MCAM functions as a competitive inhibitor of integrin-mediated migration by occupying shared binding sites on laminin α4.

## Discussion

Here we show the structural basis for MCAM recognition of laminin α4 and demonstrate that MCAM directly competes with integrin α6β1 for a shared laminin interface. Previous work established that α4-containing laminins, particularly laminin-411 and laminin-421, support cell migration through α6β1 integrin and MCAM/CD146-dependent mechanisms[15, 19]. Antibody-mapping experiments further suggested that α6β1 integrin and MCAM bind at closely apposed sites within the laminin α4 globular domain[20]. However, without a high-resolution structure of the MCAM-laminin complex, it remained unclear whether these receptors could bind laminin cooperatively or instead compete for the same physical surface. Our cryo-EM structure resolves this question by showing that MCAM engages a surface centered on the laminin α4 LG1-LG2 domains and occupies the region used by laminin-binding integrins.

Laminin-binding integrins recognize a composite T8 surface whose activity depends on the α-chain LG domains together with β- and γ-chain contributions, including the γ-chain C-terminal tail. This architecture allows integrins to interpret the broader laminin heterotrimer code, with different integrin heterodimers displaying overlapping but distinct preferences across laminin isoforms. By contrast, MCAM specifically targets the laminin α4 globular region with relative independence from β/γ-chain identity. This mode of recognition is well suited to act specifically at α4-laminin-rich environments, including vascular and perivascular basement membranes, post-capillary venules, lymphatic and tumor-associated vessels, and inflammatory barrier-crossing sites such as the neurovascular unit. In these settings, MCAM may function as a directed α4-laminin reader. Thus, MCAM may provide a mechanism for selectively engaging α4-rich vascular or stromal microenvironments while bypassing some of the β/γ-chain constraints that govern integrin binding. At the same time, because MCAM occupies a laminin α4 surface shared by integrins, MCAM binding could either promote localization to α4-laminin-rich niches or, when present in soluble or matrix-bound form, mask integrin-accessible sites and restrain integrin-driven migration.

These findings have therapeutic implications. Integrins are attractive but challenging drug targets because they are widely expressed, functionally pleiotropic and involved in normal adhesion, immunity, hemostasis and tissue homeostasis[28, 29]. Direct systemic integrin blockade can therefore produce broad and unwanted effects. The MCAM-laminin α4 structure identifies an alternative strategy: targeting the laminin α4 receptor-binding surface itself. Biologics that mimic the MCAM footprint, stabilize an MCAM-like blocked state, or use engineered MCAM-derived scaffolds could selectively prevent α6β1 integrin engagement on α4-containing laminins without globally inhibiting integrin function. Because laminin α4-containing vascular basement membranes regulate leukocyte trafficking and tumor-cell migration[6, 7, 15, 19, 20], such approaches may be relevant to metastatic dissemination, neuroinflammation and inflammatory cell trafficking.

Several limitations should be considered. The current work defines a competitive mechanism using purified proteins, structural analysis and in vitro migration assays. It does not directly quantify receptor-density thresholds, cell-surface occupancy, soluble versus membrane-tethered MCAM balance, or in vivo basement membrane occupancy. It also does not exclude additional MCAM functions mediated by the cytoplasmic tail, lateral membrane organization, endothelial interactions or soluble ectodomain signaling[16–18, 30]. Rather, the study defines a precise structural mechanism by which MCAM can inhibit laminin-dependent migration: direct competition with α6β1 integrin for laminin α4.

In summary, we show that MCAM and integrin α6β1 compete for a shared laminin α4 interface. This high-resolution structure explains how MCAM occupancy can suppress integrin-dependent migration, resolves a long-standing ambiguity in MCAM-laminin biology, and establishes a structural foundation for therapeutic strategies aimed at selectively blocking pathological cell migration.

## Materials and Methods

### Recombinant Protein Expression and Purification

Codon-optimized cDNAs encoding human MCAM (V24 – H646), laminin α4 (LamA4, N757 – E1405), laminin β1 (LamB1, D1714 – L1786), laminin β2 (LamB2, A1727 – Q1798), and laminin γ1 (LamC1, D1528 – P1609) were synthesized by IDT. The MCAM and LamA4 constructs were cloned into mammalian expression vectors containing a Wnt3 signal peptide (MEPHLLGLLLGLLLGGTRVLAG) and an N-terminal octa-histidine tag followed by a Factor Xa cleavage site (GHHHHHHHHGSSTSNGTIEGRS). The LamB1 and LamB2 constructs were cloned into a mammalian expression vector containing an IgG κ-chain signal peptide (MEFQTQVLMSLLLCMSGAAA). The LamC1 construct was cloned into mammalian expression vector containing an IgG γ-chain signal peptide and an N-terminal twin-strep tag (MKLPVLLVVLLLFTSPASSSSAWSHPQFEKGGGSGGGSGGSAWSHPQFEK). MCAM and LamA4 LG truncations and mutations were conducted by In-Fusion Snap Assembly (Takara). Full-length LM411 and LM421 were purchased from Biolamina (cat# LN411-02, LN421-02) or Acro Biosystems (cat# LAT-H5263-100ug). Integrin α6β1 was purchased from Acro Biosystems (cat# IT1-H52W7-100ug). LamA4 LG1–5 was purchased from R&D systems (cat# 7340-A4).

Recombinant proteins were expressed by transient transfection of Expi293 cells (Thermo Fisher Scientific). Conditioned medium was collected 3–4 days post-transfection and applied directly to Ni-Penta^™^ Agarose-Base Resins (Marvelgent Biosciences) by gravity flow. The resin was washed with buffer containing 15 mM imidazole, 100 mM NaCl, pH 8.0, and bound proteins were eluted with buffer containing 300 mM imidazole, 100 mM NaCl, pH 8.0. For further purification, eluates were subjected to size-exclusion chromatography on a HiLoad 26/600 Superdex 200 pg column or a Superdex 200 Increase 10/300 GL size exclusion column (Cytiva Life Sciences), equilibrated in 20 mM HEPES, 100 mM NaCl, pH 7.5. Peak fractions were analyzed by SDS-PAGE, concentrated using 10 kDa or 30 kDa Centricon spin concentrator (Millipore), and stored at 4 °C.

### Cryo-EM structure determination

The MCAM-LamA4 complex was prepared by mixing purified MCAMecd and LamA4 LG1–3 at a 1:1 molar ratio and diluting the mixture to approximately 0.3 mg/ml. Three microliters of sample were applied to glow-discharged Quantifoil holey carbon grids (R1.2/1.3 300 mesh). Grids were blotted for 6 s at 16 ℃ with 100 % humidity before plunge-freezing into liquid ethane using a Vitrobot Mark IV (FEI). Cryo-EM data were collected on a Glacios microscope equipped with a K3 summit direct electron detector (Gatan) using SerialEM software in super-resolution mode at a nominal magnification of 45,000×, corresponding to a calibrated super-resolution pixel size of 0.434 Å. Micrographs were acquired over a defocus range of −0.8 to −2.0 μm. The dose rate was set to 6.3 electrons per physical pixel per second. The exposure time of each movie was about 1.2 s, leading to a total dose of approximately 40 electrons per Å^2^, fractionated into 40 frames. A total of 7,387 images were collected for MCAM-LamA4 complex.

Image processing was performed in cryoSPARC[24] v4.3.018 following the standard workflows. During curation, micrographs with CTF-estimated resolution worse than 6 Å, average intensity greater than 50, or relative ice thickness greater than 1.2 Å were excluded. Initial particle picking was performed using blob picking, followed by several rounds of 2D classification. Particles belonging to good 2D classes were used for ab initio reconstruction to generate initial 3D volumes. Particles corresponding to the complex were then selected for Topaz[31]-based particle picking. The resulting particle set was subjected to iterative 2D classification, ab initio reconstruction, and heterogeneous refinement to obtain a high-quality particle class for final map generation. The best map was refined by non-uniform refinement[23], and mask corresponding to the compact MCAM and LamA4 LG1–3 regions were generated by volume segmentation in ChimeraX v1.11.1 followed by local refinement. Overall resolution was estimated using the gold-standard Fourier shell correlation (FSC) = 0.143 criteria and local resolution was calculated in cryoSPARC from the two half-maps.

### Model building and refinement

An initial model was built into the cryo-EM density using the Phenix Predict and Build: Cryo-EM module (version 1.21rc1-5127)[32]. The model was refined iteratively using Phenix Real-space Refinement module[33] and Coot[34]. Detailed refinement statistics and model-validation metrics are provided in Supplementary Data Table 1.

### AlphaFold models

The AlphaFold 3 Server was used to generate a model of the truncated E8-LM411-integrin α6β1 complex using residues N757 – E1405 of LAMA4 (Uniprot ID: Q16363), D1714 – L1786 of LAMB1 (Uniprot ID: P07942), D1528 – P1609 of LAMC1 (Uniprot ID: P11047), F24 – K680 of ITGA6 (Uniprot ID: P23229), and Q21 – E465 of IGB1 (Uniprot ID: P055556).

### Bio-layer Interferometry

Bio-layer Interferometry (BLI) was performed using an Octet RED96e instrument (Sartorius). ProteinA biosensors (Sartorius) were loaded with Fc fused MCAMecd to a response level of approximately 1 nm, equilibrated in working buffer (20 mM HEPES, 100 mM NaCl, pH 7.5) for 30 s, and then immersed in wells containing the indicated concentrations of T8-LM411, T8-LM421, or mutant proteins for 120 s, followed by dissociation in working buffer for 120 s. Equilibrium (plateau) responses were plotted against concentration using GraphPad Prism 10.3.1. For competition assay, 20 nM T8-LM411 was mixed with increasing concentrations of integrin α6β1 in working buffer supplemented with 1 mM MnCl_2_, 0.1 mM CaCl_2_.

### Transwell Migration Assay

Transwell migration assays were performed essentially as described previously[19]. Briefly, the underside of transwell inserts (Corning cat # 3422) was coated with LM411 (Biolamina or Acro Biosystems) or LM421 (Biolamina or Acro Biosystems) or with laminin pre-complexed with 1 μM MCAM at 37 ℃ for 2 h, then blocked with DMEM containing 0.1% BSA for 30 min. Cells were detached using Cellstripper (Corning cat# 25-056-CI) and resuspended in DMEM supplemented with 0.1% BSA medium. A total of 1 × 10^5^ cells were added to the upper chamber, and DMEM with 0.1% BSA was added to the lower chamber. After 18 hours at 37 ℃, transwell inserts were washed six times with sterile PBS, fixed with 4% paraformaldehyde (Santa Cruz cat# sc-281692) for 15 min, and stained using a hematoxylin and eosin staining kit (Abcam cat# ab245880) according to the manufacturer’s instructions. Two transwell inserts were analyzed per condition, and each experiment was repeated at least three times for statistical analysis.

### Western Blotting

Cells were washed with PBS and lysed in buffer containing 20mM Tris-HCl pH 8.0, 150mM NaCl, and 0.5% NP-40, supplemented with protease inhibitor cocktail (Cell Signaling Technology). Lysates were incubated on ice for 30 min and clarified by centrifugation at 15,000 rpm for 15 min. Protein concentrations were determined using a Bradford assay (Thermo Fisher Scientific). For detection of MCAM, integrin α6, and integrin β1, 50 μg total protein per sample was separated on 4–20% gradient gel for SDS-PAGE gels using BLUEstain 2 protein ladder (GoldBio) as a molecular-weight ladder. Proteins were transferred to PVDF membrane (EMD millipore) in transfer buffer containing 25 mM Tris, 192 mM glycine, 20% methonal (v/v) using a constant voltage of 90 V for 45 min. Membranes were blocked with 5% non-fat milk (Americo) in TBST buffer (20 mM Tris-HCl pH8.0, 150 mM NaCl, 0.1% Tween 20) for 1 h at room temperature followed by incubating with the primary antibodies for 1h at room temperature. Primary antibodies were used at the following dilutions: MCAM (Cell Signaling Technology cat# 81701), 1:1000; integrin α6 (Cell Signaling Technology cat# 3750), 1:1000; integrin β1 (Cell Signaling Technology cat# 9699T, 1:1000). Membranes were then incubated with horseradish peroxidase (HRP)-conjugated goat anti-rabbit IgG secondary antibody (Cell Signaling Technology) at 1:5000 dilution for 1 h at room temperature. The membranes were developed with Amersham ECL prime western blotting detection reagent (Cytiva Life Sciences) and chemiluminescence scanned using ChemiDoc (Bio-Rad).

## Supplementary Material

Supplement 1
