# Supplementary material for: Structural Basis for MCAM Mediated Inhibition of Laminin Dependent Cell Migration
Source: bioRxiv. 2026 Sep 18:2026.09.11.751044. Preprint. [Version 1] doi: 10.64898/2026.09.11.751044 (PMC13596217; doi:10.64898/2026.09.11.751044)
Supplement: Supplementary file 1 [file NIHPP2026.09.11.751044v1-supplement-1.pdf]

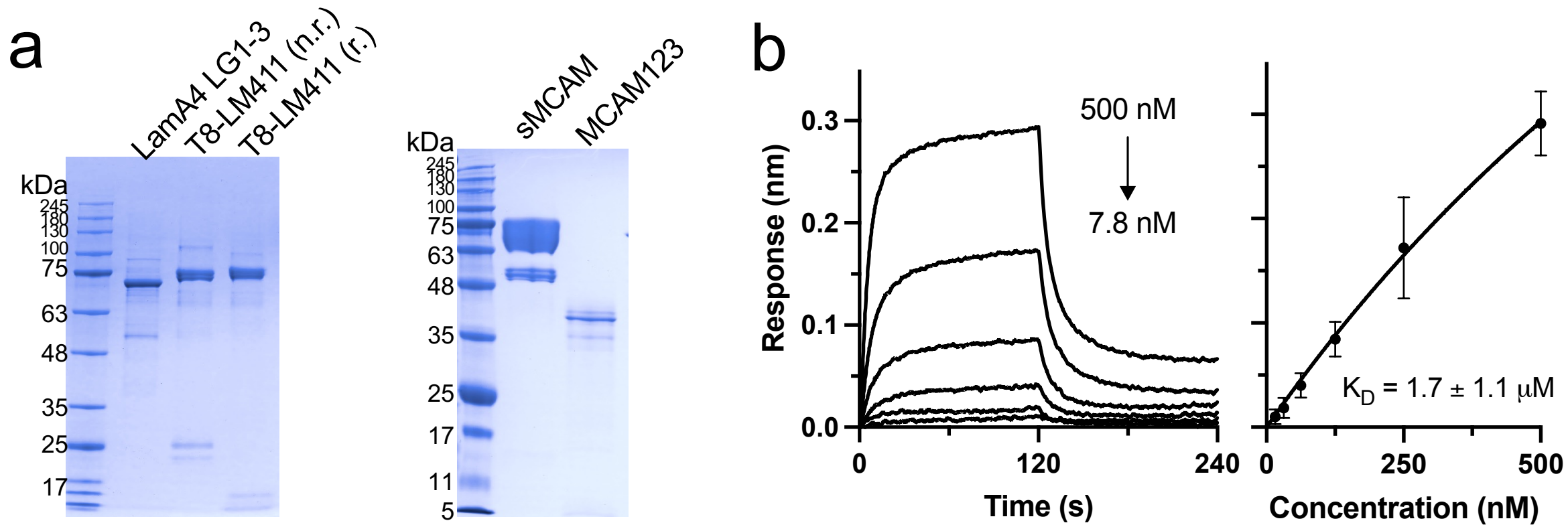

Supplementary Figure S1. Recombinant LamA4 and MCAM proteins. a, SDS-PAGE gel stained with Coomassie blue shows the sizes of different constructs of LamA4 and MCAM expressed. **b,** Direct binding of LamA4 LG1-3 to sMCAM detected by biolayer interferometry (BLI). The sensors were loaded with sMCAM. The binding affinity represents mean ± SEM from three independent experiments.

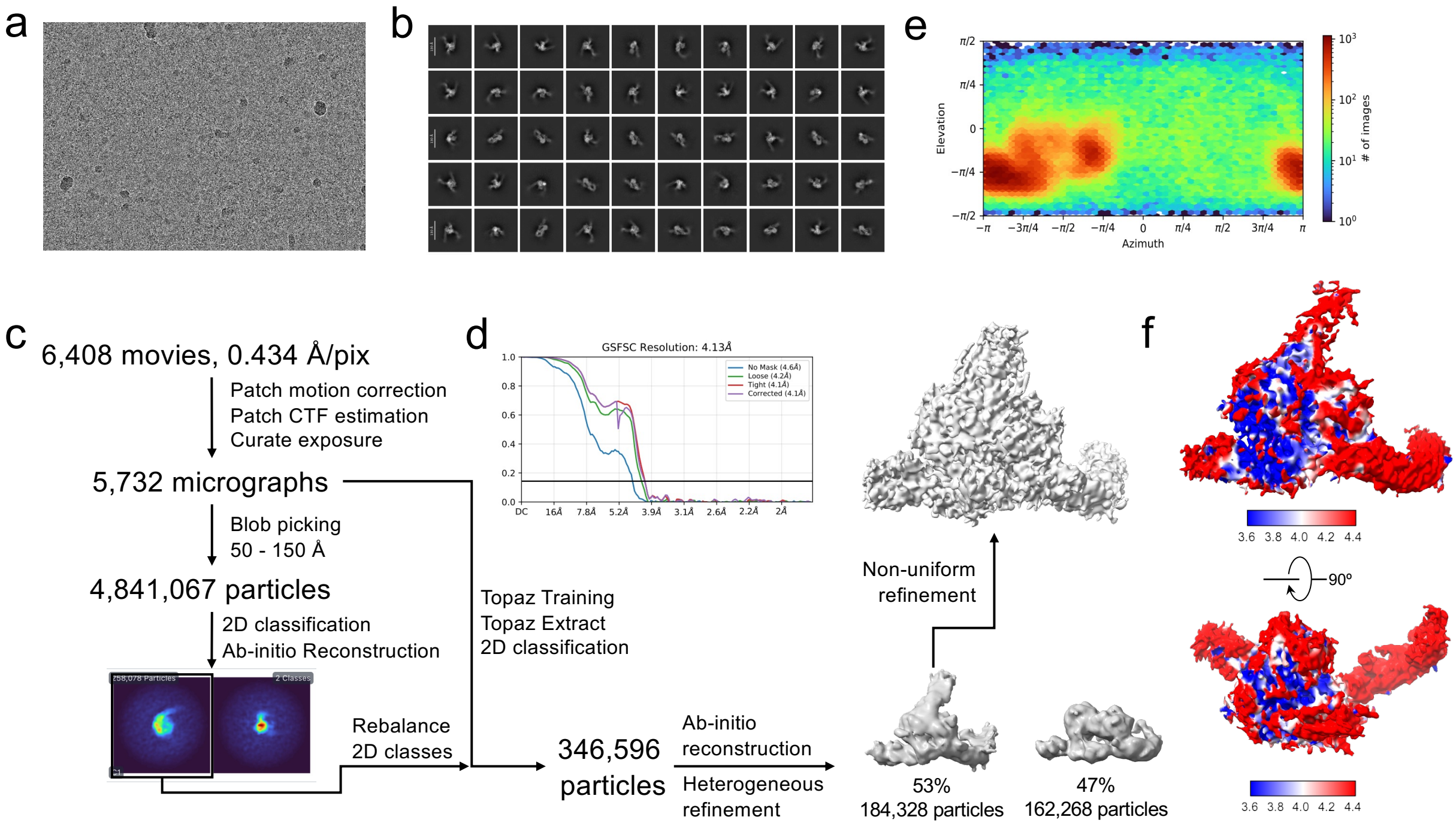

Supplementary Fig 2. Cryo-EM data processing for the MCAM-T8-LM411 complex structure. a, Representative raw micrograph of sMCAM-T8-LM411 complex. **b,** Representative 2D class averages of sMCAM-T8-LM411 complex. **c,** Images of the processing workflow for structure determination of sMCAM-T8-LM411 complex. **d,** Gold-standard Fourier Shell Correlation (FSC) of the local-refined map. **e,** Angular distribution of particles in the final map obtained by non-uniform refinement. **f,** Local resolution estimation of the map.

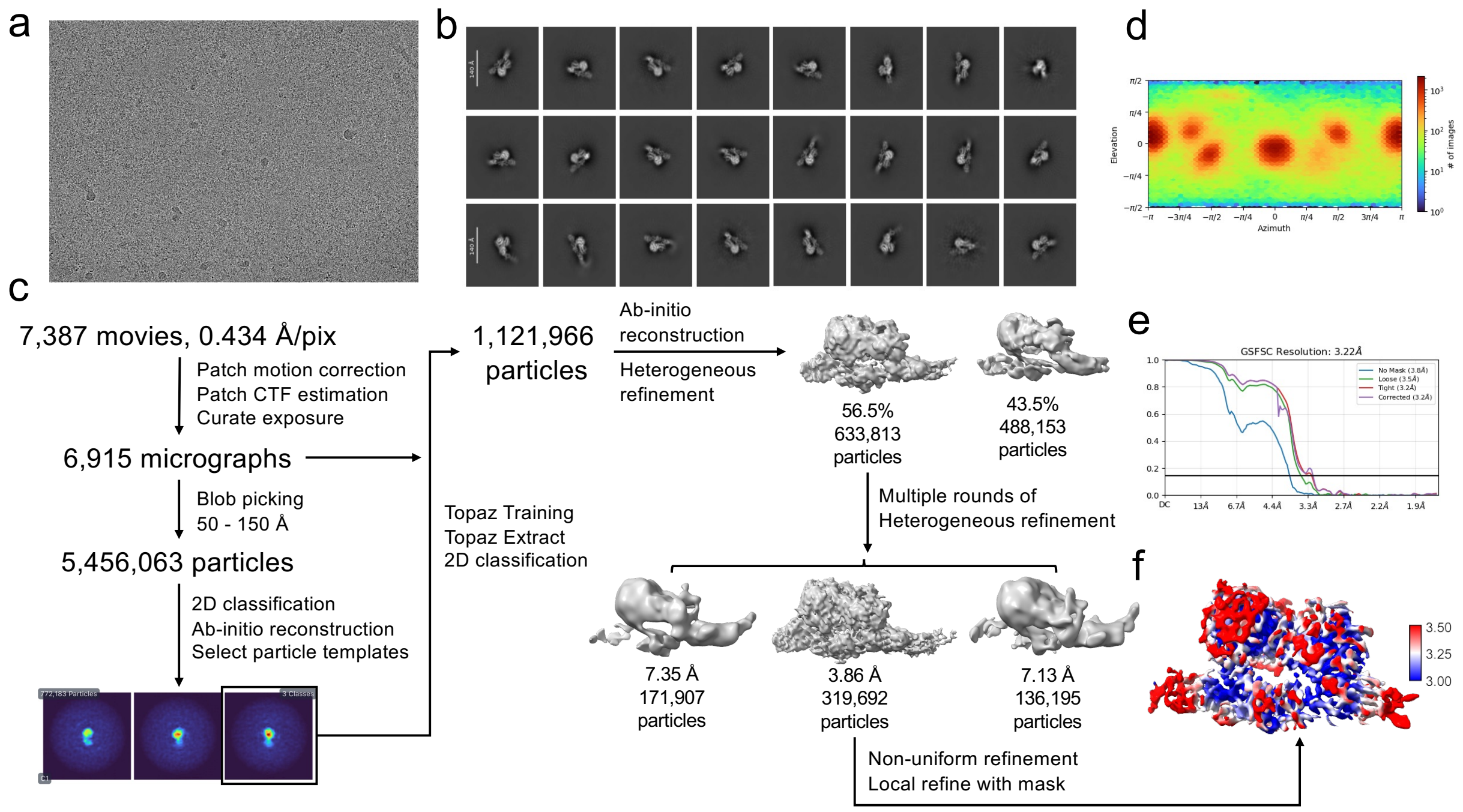

Supplementary Fig 3. Cryo-EM data processing for the MCAM-LamA4 LG1-3 complex structure. a, Representative raw micrograph of sMCAM-LamA4 LG1-3 complex. **b,** Representative 2D class averages of sMCAM-LamA4 LG1-3 complex. **c,** Images of the processing workflow for structure determination of sMCAM-LamA4 LG1-3 complex. **d,** Angular distribution of particles in the final map obtained by non-uniform refinement. **e,** Gold-standard Fourier Shell Correlation (FSC) of the local-refined map. **f,** Local resolution estimation of the map.

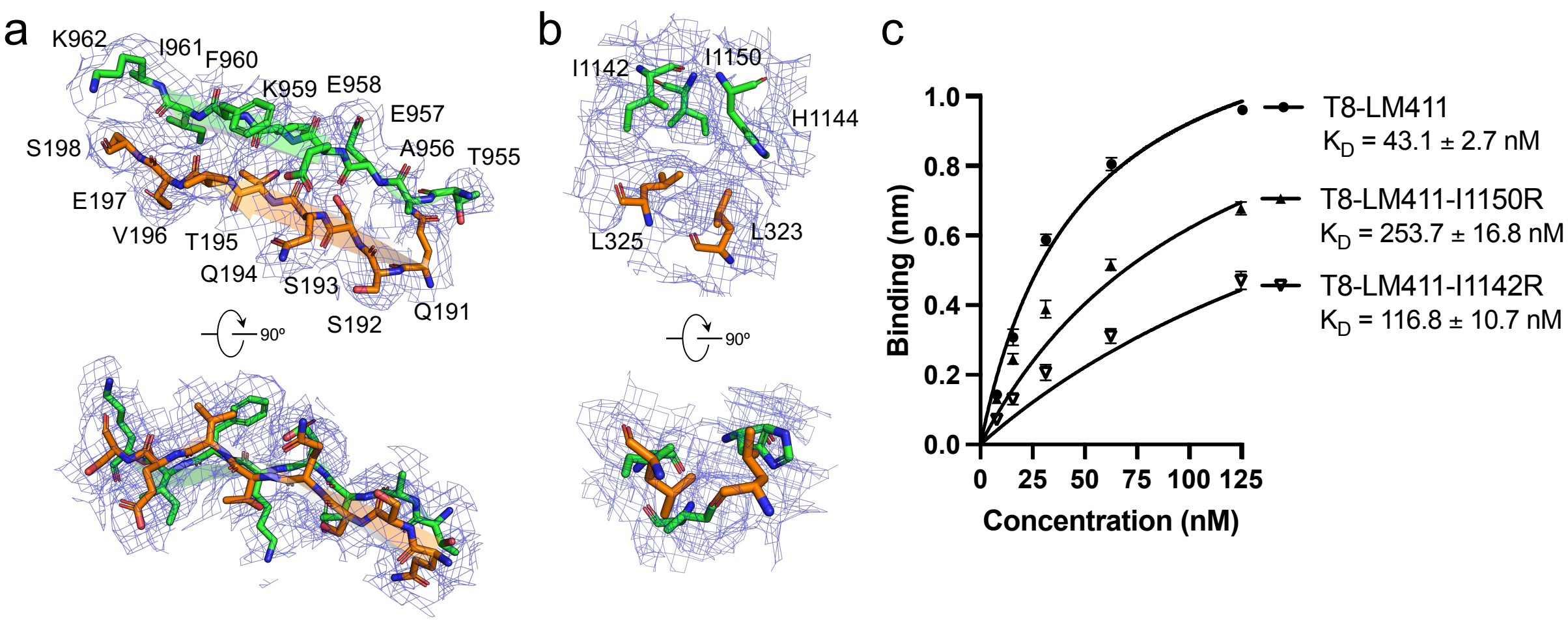

Supplementary Fig 4. Regional density map of MCAM-LamA4 interaction sites. a, Density map of MCAM-LamA4 interaction site1. **b,** Density map of MCAM-LamA4 interaction site2. **c,** Binding of T8-LM411 mutants to MCAM detected by biolayer interferometry (BLI). The sensors were loaded with sMCAM. The binding affinity represents mean ± SEM from three independent experiments.

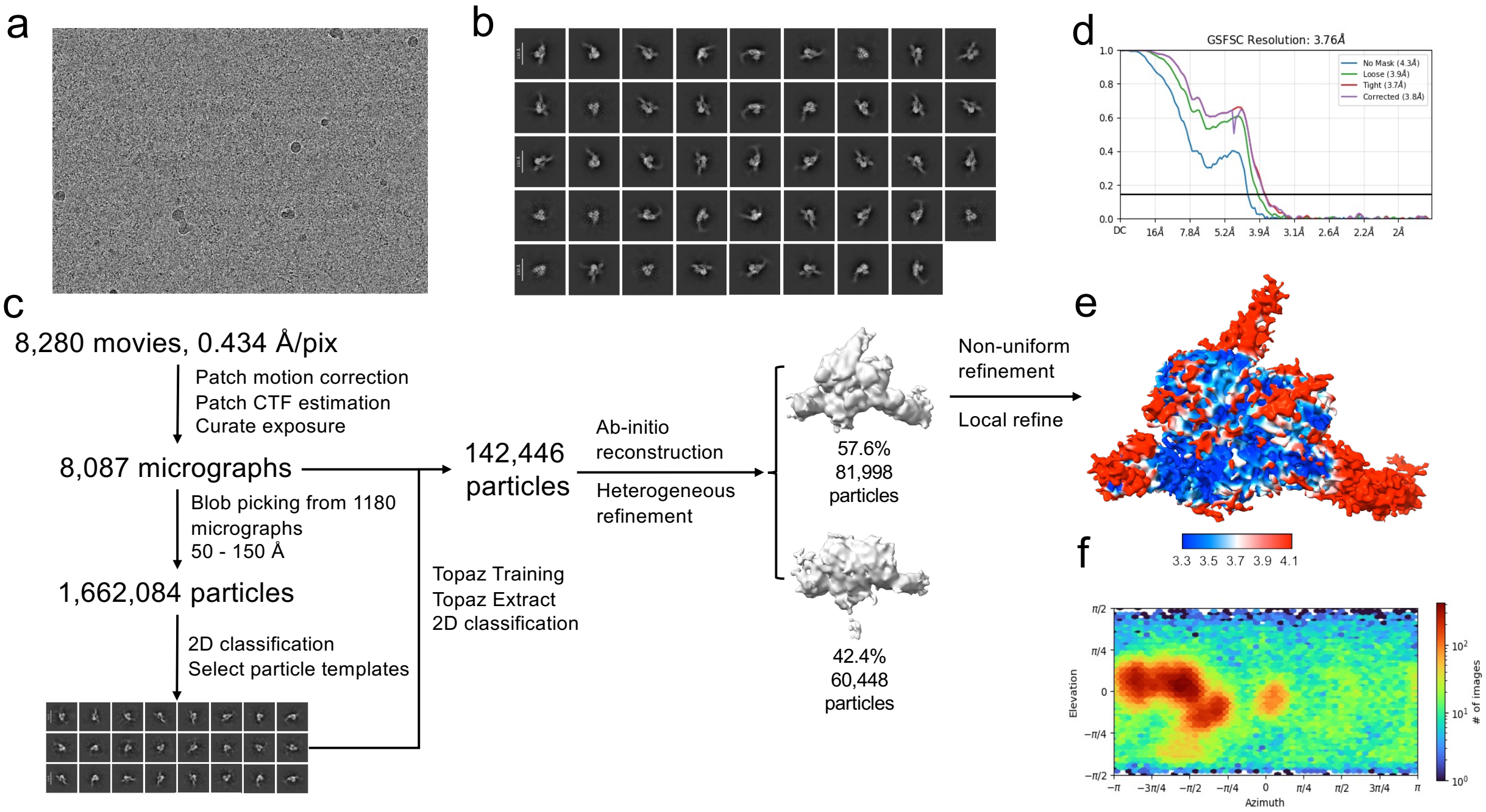

Supplementary Fig 5. Cryo-EM data processing for the MCAM-T8-LM421 complex structure. a, Representative raw micrograph of sMCAM-T8-LM421 complex. **b,** Representative 2D class averages of sMCAM-T8-LM421 complex. **c,** Images of the processing workflow for structure determination of MCAM-T8-LM421 complex. **d,** Gold-standard Fourier Shell Correlation (FSC) of the local-refined map. **e,** Local resolution estimation of the map. f, Angular distribution of particles in the final map obtained by non-uniform refinement.

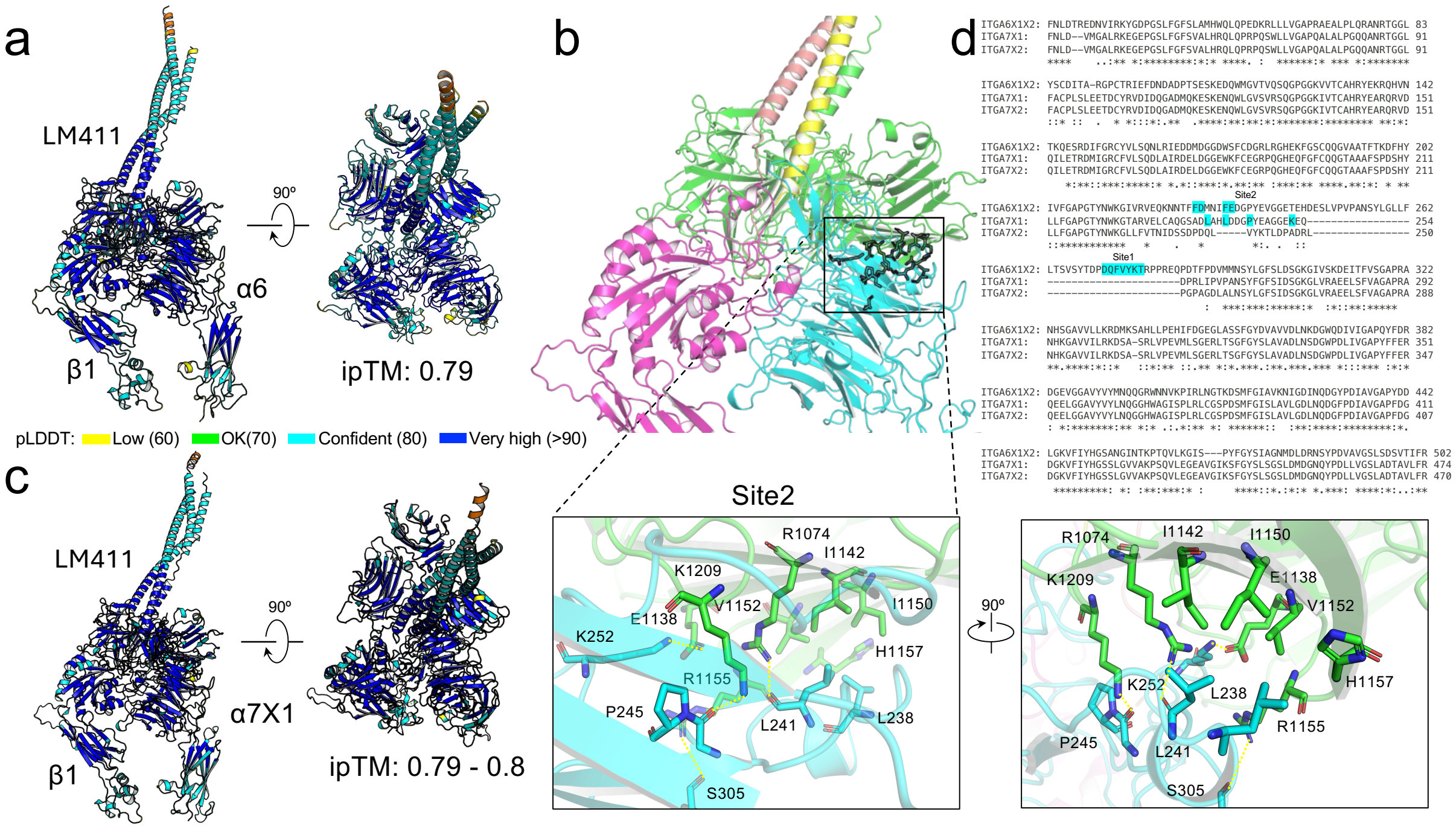

Supplementary Fig 6. AlphaFold 3 predicted structural models of integrins-T8-LM411 complex. a, pLDDT plot of AlphaFold 3 predicted integrin α6β1-T8LM411 complex structure and its ipTM. **b,** AlphaFold 3 predicted structural model of T8-LM411 and integrin α7X1β1 complex and the enlarged detailed view of site2 interactions between integrin α7X1 and LamA4 LG2 domain. **c,** pLDDT plot of AlphaFold 3 predicted integrin α7X1β1-T8LM411 complex structure and its ipTM. **d,** sequence alignment among integrin α6X1X2, α7X1 and α7X2. Residues highlighted in cyan are predicted to interact with LamA4.

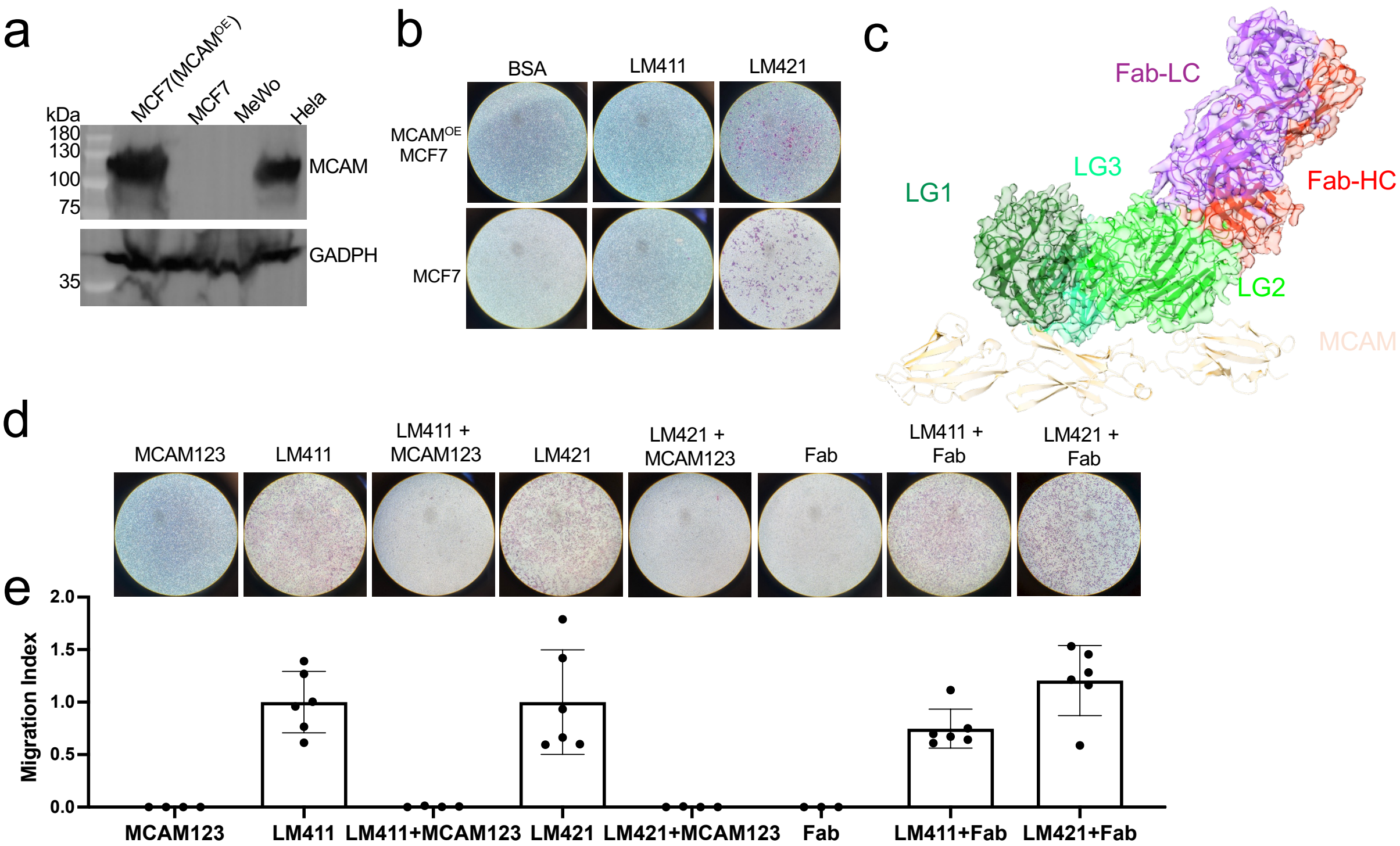

Supplementary Fig 7. Laminin α4 promotes cell migration primarily through integrin-dependent mechanisms. a, Western blot analysis of MCAM protein expression in MCF7 breast cancer cells stably overexpressing full-length MCAM, parental MCF7 cells, and MeWo melanoma cells, with Hela cells included as a positive control. **b,** Overexpression of full-length MCAM in MCF7 breast cancer cells doesn't endow MCF7 with enhanced migration towards LM411 and LM421. The concentration of LM411 and LM421 is 250 nM. **c,** Surface view of the Cryo-EM reconstruction (3.52 Å, Supplementary Fig. 8) and ribbon diagram of an atomic model of LamA4 LG1-3-Fab complex. MCAM is shown in cartoon representation to illustrate the location of the Fab epitope relative to the MCAM binding interface. **d,** The first three Ig-like domains of MCAM, i.e. MCAM123, which engage in LamA4, are sufficient to inhibit Laminin α4-mediated MeWo melanoma cell migration. In contrast, the Fab recognizing the LG2 domain of LamA4 at an epitope distinct from the MCAM/integrin binding site does not inhibit LamA4-driven MeWo cell migration. The concentrations of LM411 and LM421 are 100 nM and 25 nM, respectively. The concentration of MCAM123 and Fab is 1 µM. e, Quantification of the migration assays shown in d. Data are compiled from at least three independent experiments.

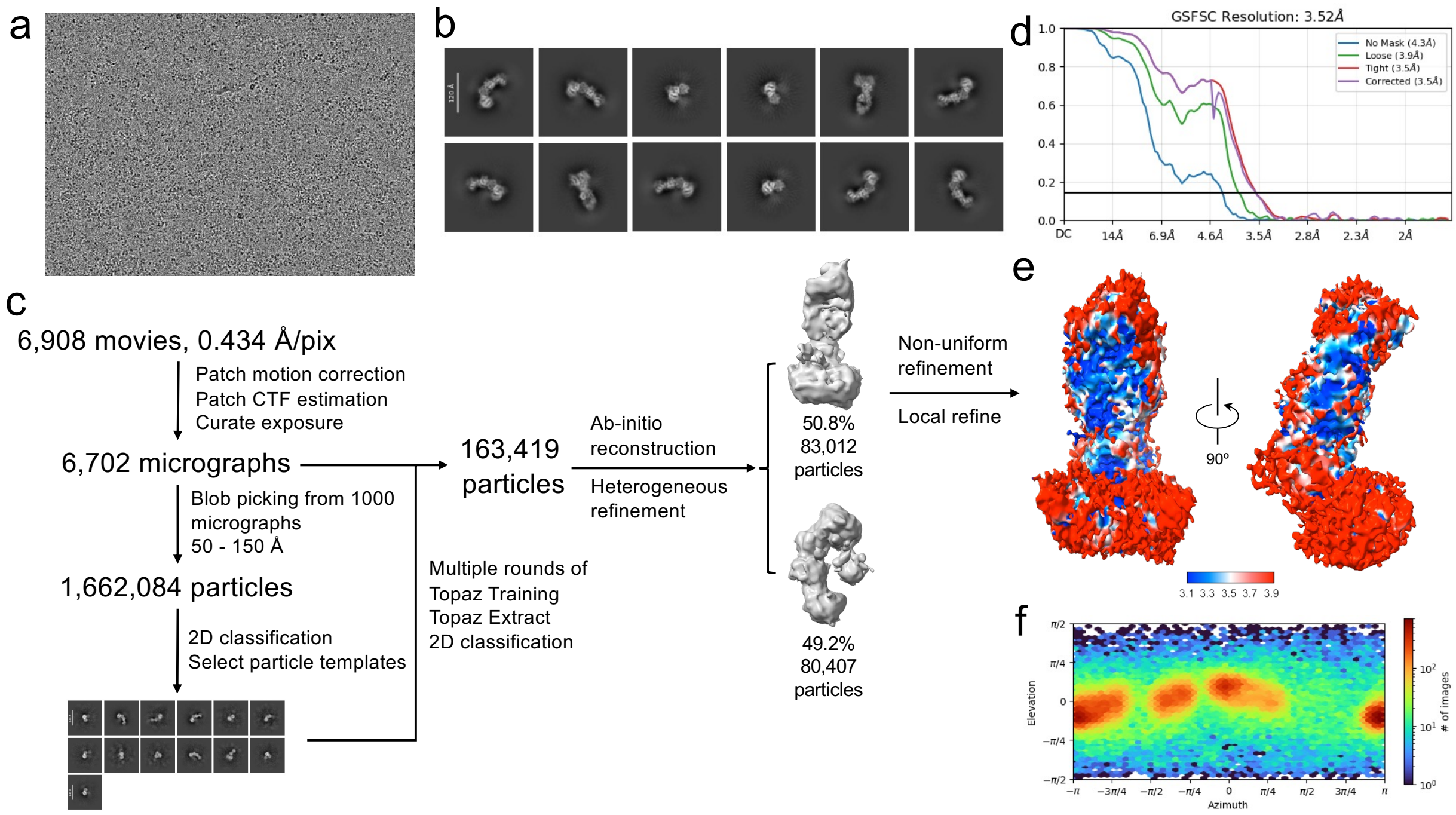

Supplementary Fig 8. Cryo-EM data processing for the LamA4 LG1-3-Fab complex structure. a, Representative raw micrograph of LamA4 LG1-3-Fab complex. **b,** Representative 2D class averages of LamA4 LG1-3-Fab complex. **c,** Images of the processing workflow for structure determination of LamA4 LG1-3-Fab complex. **d,** Gold-standard Fourier Shell Correlation (FSC) of the local-refined map. **e,** Local resolution estimation of the map. **f,** Angular distribution of particles in the final map obtained by non-uniform refinement.

Supplementary Data Table 1 l Cryo-EM data collection, refinement and validation statistics

	sMCAM – T8-LM411	sMCAM – LamA4 LG1-3	sMCAM – T8-LM421
**Data collection**			
Microsope	Glacios	Glacios	Glacios
Voltage (kV)	200	200	200
Magnification	45,000	45,000	45,000
Detector	K3	K3	K3
Electron exposure (e^-/ Å^2)	40.87	40.2	40.95
Defocus range (μm)	-0.8 to -2.0	-0.8 to -2.0	-0.8 to -2.0
Pixel size (Å/pixel)	0.434	0.434	0.434
**Cryo-EM reconstruction**			
Particles refined	184,328	319,692	81,998
Resolution (Å)	4.13	3.22	3.76
FSC threshold	0.143	0.143	0.143
Symmetry imposed	C1	C1	C1
**Refinement**			
No. of non-hydrogen atoms	7778	6739	7932
Protein residues	978	847	998
MolProbity score	2.32	2.16	2.03
Clashscore	20.23	18.57	13.06
R.m.s. deviations			
Bond lengths (Å)	0.003	0.004	0.003
Bond angles (°)	0.713	0.710	0.651
Ramachandran analysis			
Favored (%)	90.89	94.10	93.93
Allowed (%)	9.01	5.78	6.07
Outlier (%)	0.1	0.12	0.00
Rotamer outlier (%)	0.00	0.4	0.34
